# Representation of Substance Use Issues in Old Age Within Norwegian Policy Frameworks

**DOI:** 10.1177/14550725261462004

**Published:** 2026-07-01

**Authors:** Gloria Abena Ampim, Gudmund Ågotnes

**Affiliations:** 1 Department of Welfare and Participation, Western Norway University of Applied Sciences, Bergen, Norway

**Keywords:** substance use, older adults, Norway, discourse analysis

## Abstract

**Aims:**

This study examines how national policies have framed substance use issues among older adults in Norway.

**Methods:**

An analysis of 18 Norwegian white papers published between 2003 and 2024 by the Ministry of Health and Care Services, the Ministry of Social Affairs, and related ministries was conducted through “What Is the Problem Represented to Be?” poststructural analysis.

**Results:**

White papers acknowledge increasing alcohol and substance use among older adults and depict these developments as significant challenges for health and social services both at present and in the future. In response, the following three strategies are proposed: promoting active aging to address inactivity, which can contribute to substance use; combating loneliness and isolation, which represent both the causes and consequences of substance use among elderly individuals; and implementing preventive home visits to help older individuals manage daily challenges and recognize early signs of harmful substance use.

**Conclusions:**

Relevant policies primarily address alcohol and prescription drugs but largely ignore the so-called illicit substances. The strategies employed constitute a funnel, leading from broad and preventive strategies to narrow and curative strategies. Although this variation holds some promise, we argue that these policies inadvertently portray substance use in older adults as if all individuals with substance use problems share the same characteristics, experiences, and needs. This generalization risks exacerbating health inequalities among older adults who use substances.

## Introduction

Over the past two decades, research has consistently documented a significant increase in the use of alcohol, tobacco, cannabis, opioid maintenance treatment (OMT), and prescription drugs with addictive potential among people aged 50 years and older (Benshoff et al., 2003; Flint et al., 2018; Kuerbis, 2019; Mattson et al., 2017; Kaitala et al., 2026). The baby boom generation, which has been exposed to a wide range of legal and illegal substances across the life course, is now entering older age and constitutes a new cohort of older people (Chhatre et al., 2017; Kuerbis & Sacco, 2013). Globally, the most commonly used substances in this age group are alcohol, cannabis, and opioids (Kuerbis, 2019, p. 1).

Alcohol is especially harmful in older age due to age-related physiological changes, slower metabolism, and increased sensitivity to alcohol, all of which heighten the risk of adverse events such as falls and injuries (Bergh et al., 2021; Kelly et al., 2018; Patra et al., 2024; Rondestvedt, 2024). Older adults’ increased alcohol use has coincided with a rise in their intake of addictive prescription drugs, a combination that can lead to negative health outcomes (Bergh et al., 2021; Rondestvedt, 2024). Consequently, older adults who use substances are more likely to experience poorer health than their peers who do not use substances (Bjerge et al., 2024).

In addition, older adults are less likely to seek help for substance use–related problems and may feel shame about disclosing their struggles (Lunde, 2013). Caregivers also commonly perceive alcohol use among older people as a late-life indulgence and may avoid conversations about substance use in order not to offend them (Bareham et al., 2020). Some Norwegian nursing homes, for example, primarily regard alcohol as a cultural symbol linked to quality of life, and, in the absence of clear institutional policies on alcohol use, practices often vary considerably (Johannessen et al., 2021).

Furthermore, gaps in health professionals’ training and expertise in diagnosing and treating substance use among older adults, combined with weak interservice coordination and referral pathways, compromise both access to and the quality of treatment for this population (Blanco & Lennon, 2020; Chhatre et al., 2017; Flesland, 2014; Johannessen et al., 2021; Kuerbis, 2019; Lunde, 2013). Limited diagnostic and treatment options, together with reluctance to engage in open dialogue, help perpetuate the lack of awareness and recognition of alcohol and substance use problems in this demographic (Benshoff et al., 2003; Beynon, 2009; Chhatre et al., 2017; Kuerbis, 2019).

The challenges outlined in the literature support researchers’ calls for tailored guidelines and services that address the social determinants of health in older adults (Beynon, 2009; Blanco & Lennon, 2020; Chhatre et al., 2017). These studies argue for the development of integrated, multidisciplinary approaches to prevent and treat substance use problems in this population.

### Policy and Research Gaps in Older Adults' Substance Use

Policy-making, interventions, and research have often failed to address the specific needs and challenges associated with substance use in later life (Beynon, 2009; Blanco & Lennon, 2020; Bjerge et al., 2024; Zechner et al. 2024). The focus on heavy episodic drinking and more incidences of intoxication among younger drinkers (Kuerbis & Sacco, 2013), who are often perceived as more able than older adults to change their substance using habits and benefit from treatment (Lunde, 2013), has contributed to a relative neglect of older adults (Beynon, 2009). Yet older adults’ risk may be substantial even at moderate levels of alcohol and drug consumption, given the physiological and clinical factors outlined above.

Nevertheless, in Norway, the analysis by Bye and Moan (2020) suggests that recent years have seen a leveling out of differences between younger and older adults in the proportion who drink frequently and in a risky manner. Among older adults (60 years and older) in Norway, alcohol consumption has increased substantially over time, with the proportion who drink rising from 60% in 1985 to 80% in 2005 (Bye & Moan, 2020, p. 448). Over the same period, women have increasingly been drinking as much as men, thereby narrowing the traditional gender gap in substance use (Fredwall & Antonsen, 2023; Stelander et al., 2021). An exploration of motivations for alcohol use among older adults found that coping with negative feelings was a key driver of both “normal” and harmful drinking (Santora et al., 2022). In addition, longitudinal evidence demonstrates that older adults (65 years and older) who consume alcohol more than 4 days a week are more likely than younger adults to be prescribed drugs with addictive potential (Tevik et al., 2019).

A coinciding demographic development is that the number of people aged 65 years or older in Norway is projected to double over the next 50 years (Blix & Ågotnes, 2023). The rising number of older adults who drink alcohol in Norway is likely to lead to an increase in alcohol-related problems within this group (Bye & Moan, 2020; Fredwall & Antonsen, 2023; Rondestvedt, 2024). A growing older population, combined with rising and changing patterns of substance use (Fredwall & Antonsen, 2023), raises critical questions about how well the welfare state is prepared – particularly at the policy level – to meet these needs. In particular, how are older adults’ substance use problems conceptualized in national policies? Which policy instruments and solutions are employed, and what kinds of “problems” are they designed to address.

### Aims of the Present Study

The present study does not seek to define what constitutes substance use among older people or to explain why its prevalence is rising. Rather, we focus on how the issue is constructed in policy, with particular attention to the Norwegian context. Specifically, we examine national-level guidelines and strategies concerning substance use among older adults, aiming to improve our understanding of how these policies respond to what is increasingly recognized as a “rising challenge.” The study has three main objectives:

1. To identify the key national strategies and policies for addressing substance use among older adults in Norway.
2. To analyze how these policies define and frame “the problem” of substance use in old age.
3. To discuss potential implications of these policy framings for the organization and delivery of care to older people.

Inspired by governmentality perspectives associated with Foucault (1991) and subsequent work (Lemke, 2002), we pay particular attention to how policies across health and social sectors may be interconnected and how they interact with broader socioeconomic discourses. In doing so, we seek to illuminate how national policy frameworks shape the management of substance use among older adults and with what possible consequences for equity and care in later life.

### Governmentality

The contemporary scholarship on governmentality can be traced back to Foucault's notion of government and governmentality introduced in 1978 to further his analyses of power relations (Danaher et al., 2000; Lorenzini, 2023; Nigro et al., 2014, p. 130). Foucault explained government as “the conduct of conduct,” which involves “governing the self” and “governing others” (Lemke, 2002, pp. 50–51). Governmentality is thus an attempt to explore the reasoning and actions of different forms of government in their attempts to manage the individual, social, economic, religious, medical, philosophical, and political lives of others, a group or a population (Lemke, 2002; Rose & Miller, 2010). In this study, we explore how national programmes attempt to manage and conduct the conduct of older adults through strategies aimed at reducing harmful substance use in old age.

Following a governmentality perspective, we attempt to elucidate the role of political knowledge in justifying what is considered “good, healthy, normal, virtuous, efficient, or profitable” in shaping the lives of older adults (Foucault, 1991; Lemke, 2002; Rose & Miller, 2010, p. 175). Accordingly, policies, programmes, projects, and plans – which Rose & Miller (2010) call political rationalities – are not neutral entities existing outside social relations; rather, they emerge from and are enacted within those relations (Rose & Miller, 2010, p. 175). Political rationalities are developed in relation to particular understandings of their objects of governance, such as society, the nation, the population (in this context, older adults), and the economy (Bacchi, 2016; Ball, 2015; Rose & Miller, 2010, p. 179).

In the context of substance use among older adults, it is therefore useful to analyze how governmental strategies frame the problem of late-life substance use and to unpack the discursive mechanisms that present these strategies as authoritative “politics of truth” (Lemke, 2002, p. 55). How governments conceptualize both older adults and substance use shapes the types of interventions that are proposed. These framed interventions, in turn, could produce new forms of knowledge that influence institutional practices in older-age care and substance-use services.

## Methods

### What is the Problem Represented to Be (WPR)?

The WPR approach links knowledge to power and politics on the basis of the understanding that knowledge is a contested form of political creation (Bacchi, 2016, 2018, 2023). Unlike the analysis of debates and arguments through language use carried out in critical discourse analysis (Fairclough, 2003), WPR performs what Bacchi (2018, para 4) describes as an “analysis of discourses (knowledges)”. The task of WPR is not to inquire how governments solve problems from an external vantage point but to pursue critical investigations of how governmental (personal, social and institutional) practices articulate particular kinds of problems (Bacchi & Goodwin, 2016, p. 14).

According to this approach, the process of policy making involves decision-making and choosing from alternatives whereby some choices become known and approved, whereas others remain obscured. Using WPR, researchers are encouraged to move away from “problem-solving” to “problem-questioning” in policy research to invigorate the discussion of how and why some alternatives become approved, whereas other alternatives remain disguised (Riemann, 2023; Tawell & McCluskey, 2022). Instead of the regular method of starting with the analysis of problems, WPR begins with an analysis of the solutions and investigates specific kinds of problems that are implied in so-called solutions, strategies, directives, and recommendations (Bacchi & Goodwin, 2016). Notably, acclaimed official “problems” for which policy makers develop solutions are not necessarily the reasoning behind policies, regulations, and recommendations.

To perform WPR analysis, Bacchi (2016, p. 9) suggested six interrelated questions as guidelines for researchers. The first question inspires researchers to ask about the problem definition in a specific policy. The second question suggests an interrogation of the presuppositions and assumptions underlying the specific representation of the problem. The third question relates to how the representation of the problem has occurred, an analysis that involves a form of Foucauldian genealogy. The fourth question asks what has been treated as unproblematic and what possible assumptions make it difficult to make alternative choices. Question five explores the effects and types of subjects, discursive practices, and lived experiences that specific approaches to representing a problem could produce. The sixth and final question in the WPR analysis highlights how and where this problem representation has been produced, disseminated, and defended and what possibilities for disruption and reformulation are available.

Researchers have demonstrated the effectiveness of the WPR methodology as a critical approach that can reveal how policies are shaped by ongoing political discourses and that can offer multifaceted interpretations rather than presenting policies as straightforward, rational, and objective solutions to societal issues (Pringle, 2019; Riemann, 2023; Woo, 2023). This approach is not prescriptive but rather iterative, and researchers are at liberty to choose which questions to answer and in what order (Bacchi & Goodwin, 2016; Riemann, 2023). In this study, we focus on the following three key WPR questions: Q1, “What is the ‘problem’ represented to be in policies designed to address substance use among older adults?”; Q2, “What presuppositions and assumptions underlie this representation of the ‘problem?’”; and Q5, “What effects are produced by this representation of the ‘problem?’” (Bacchi, 2016, p. 9). We believe that addressing these three questions will enable us to better understand the complexities and implications of policies and subsequent strategies related to substance use within this demographic. Importantly, these questions are not examined in isolation or sequentially; rather, they are approached in a mutually reinforcing manner.

### Data Selection and Analysis

The data on which this study focuses have been drawn from national-level documents in the form of white papers, which have been acknowledged as key political frameworks for forecasting Norwegian elderly care policies (Christensen & Fluge, 2016). The topic of substance use among older adults lacks dedicated white papers; instead, it is typically addressed within broader policy frameworks related to public health, elder care, and national strategies concerning alcohol and substance use. This position is reflective of the complex nature of substance use issues, which require the collaboration of various government agencies. Moreover, services related to elder care are particularly fragmented and vertically divided among different governmental structures (Vabø et al., 2022), suggesting that the approach to addressing substance use among older adults follows a similarly disjointed pattern.

In 2003, a major proposal to reform drug use was suggested, including the reorganization of social and care services for people experiencing substance use problems (Norwegian Ministry of Social Affairs, 2003). We consider that this reform represented a pivotal shift in Norway's national response to substance use. As a result, we decided to review white papers published after this reform, from 2003 to 2024, that make reference to aging and/or problems and solutions related to substance use in old age. Eighteen white papers were included, stored in NVivo (https://lumivero.com/products/nvivo) and coded using numerous search terms such as “rus” (drugs/substance), “alkohol” (alcohol), “eldre” (elders), “aktiv aldering” (active aging), “ensomhet” (loneliness), “psykisk helse” (mental health), and “frivillig”/“frivillighet” (volunteering). In the second phase, all the codes were transferred from the NVivo project and subsequently, translated into English using DeepL (https://www.deepl.com), an AI-generated translation tool. These translated versions of the codes were read multiple times to gain an overview of key issues related to substance use among elderly people. To present a clearer chronological overview, an Excel document (Microsoft Corp,) was created that organized the codes into the following three distinct timelines: 2003–2012; 2013–2020; and 2020 to present. This compilation provided an overview of significant cooccurring developments related to elderly care, aging, substance use, and public health. The process identified seven white papers that provided somewhat extensive coverage of substance use among older adults, whereas 11 that did not address the issue in sufficient depth were excluded. However, those excluded papers are referenced intermittently to substantiate the comments made in the seven included papers. An overview of the seven documents included in this analysis is presented in Table 1.

**Table 1.** Norwegian White Papers Included in the Analysis.

White paper number/year of publication	Ministry	English title	Norwegian title
Prop. 15S 2015	Ministry of Health and Care Services	The Escalation Plan for the Substance Use Field 2016–2020	Opptrappingsplanen for rusfeltet 2016–2020
Meld St. 15 2018	Ministry of Health and Care Services	A Full Life – All Your Life. A Quality Reform for Older Persons	Leve hele livet. En kvalitetsreform for eldre
Meld St.19 2019		Public Health Report: Good Lives in a Safe Society	Folkehelsemeldinga: Gode liv i eit trygt samfunn
2021	The Ministries	National Alcohol Strategy. An Alcohol Policy that Promotes Health and Solidarity 2021–2025	Nasjonal alkoholstrategi. En helsefremmende og solidarisk alkoholpolitikk 2021–2025
Meld St. 15 2023	Ministry of Health and Care Services	Public Health Report: National Strategy for Levelling Out Social Inequalities in Health	Folkehelsemeldinga: Nasjonal strategi for utjamning av sosiale helseforskjellar
Meld St.23 2023	Ministry of Health and Care Services	Escalation Plan for Mental Health (2023–2033)	Opptrappingsplan for psykisk helse (2023–2033)
Meld St.24 2023	Ministry of Health and Care Services	Community and Mastery. Stay Safe at Home	Fellesskap og meistring. Bu trygt heime

## Results

Following the identification of relevant documents for inclusion, we searched for policies that address substance use issues in old age as well as more or less specific strategies for approaching “the problem.” Although reports from 2003, 2012 and 2014 briefly mentioned the increase in substance use in old age, “the Escalation Plan for the Substance Use Field 2016–2020” (hereafter referred to as the Plan) published in 2015 marked the starting point for addressing the theme in more detail. Later documents appear to have been influenced by the “problem definition” from the Plan, summarized as follows:

Alcohol use in combination with poor nutritional status and high consumption of pharmaceuticals increases the risk of illness, accidents and alcohol-related health and social problems among elderly people. Both demographics and changing drinking habits in new generations of older persons are likely to create major challenges for health, care and social services in the years to come, both in terms of capacity and in terms of how services should be designed to meet the needs of older persons. The growth and extent of older persons’ alcohol consumption, often in combination with a high consumption of medication, requires us to pay increased attention to older persons and alcohol in the years ahead. This applies to research, prevention and treatment. (Norwegian Ministry of Health and Care Services, 2015, p. 18)

According to the Plan, the identified areas of need included increasing awareness among elderly people on the effects of harmful substance use, increasing screening opportunities in the municipalities, and increasing the competence of health care services to deliver appropriate care. In other words, the Plan highlights the increased use of alcohol among older adults and the problematic interactions with (high) use of prescription drugs, which increasingly burden health care services. Following this problem description and needs assessment, three specific strategies were proposed. The first recommendation was preventive home visits to enhance older individuals’ ability to manage daily challenges, identify early signs of harmful substance use, and provide timely assistance. The second strategy emphasized “mobilization against loneliness” among elderly individuals, whereas the third focused on a “modern aging strategy” that prioritizes active aging (Norwegian Ministry of Health and Care Services, 2015, pp. 38–39).

Subsequent white papers published between 2017 and 2024 have consistently referred to these three strategies as essential for addressing harmful substance use among older adults. The 2018 white paper on reformulating policy for older people sets its target population as persons aged 65 years and older (Norwegian Ministry of Health and Care Services, 2018, p. 10). On this basis, we examine the three approaches as the principal narratives shaping substance-use strategies directed at people in this age group. We seek to understand how substance use among older individuals is framed and characterized within these three proposed solutions.

### Promoting Active Aging

“Active aging” is increasingly viewed in white papers, both implicitly and explicitly, as a vital strategy in addressing substance use problems among elderly people. This strategy is grounded in the premise that inactivity can lead to a range of harmful behaviors, including substance use. Inactive individuals may experience feelings of loneliness, boredom or depression, all of which can increase the likelihood of turning to alcohol or drugs as a means of escape (Norwegian Ministry of Health and Care Services, 2015). Moreover, the concept of active aging encompasses not only physical activity, but also mental stimulation and social interaction. Therefore, promoting active aging is seen as a means to mitigate these risks, especially during the transition from work to retirement. The argument is summarized as follows in the Plan under the section “Strategy for a Modern Elderly Policy”:

With good lifestyle habits such as social, physical, and cognitive activity, as well as good nutrition, older people can enjoy more healthy years of life. In this context, it is important to monitor developments in older people's use of alcohol and other intoxicants, perhaps especially in the transition from working life to retirement. Excessive use of intoxicants may reduce older people's opportunities to be active and maintain good health and coping skills. (Norwegian Ministry of Health and Care Services, 2015, p. 39)

In this context, challenges related to substance use are portrayed as contrary to the ideal of “active aging.” Substance use not only jeopardizes the health and well-being of older adults but also diminishes their potential to enjoy healthier, more fulfilling years. This understanding of active aging was already presented in the 2014 white paper on public health, which points out the valuable contributions that older individuals can make to both the workforce and social life (Norwegian Ministry of Health and Care Services, 2014, p. 10). Thus, the negative impact of substance use not only affects individual well-being, but also prevents older individuals from engaging fully in community life and from taking full advantage of their ability to achieve collective benefits.

In the 2018 policy for older adults titled “A full life – all your life” (“Leve hele livet”), published by the Ministry of Health and Care Services, *active* older people are further addressed in relation to mental health in public health work and facilitating health-friendly choices (Norwegian Ministry of Health and Care Services, 2018). In the white paper on “Community and Mastery” (“Fellesskap og Meistring”), published by the same ministry, the approach to active aging is advanced with several key objectives (Norwegian Ministry of Health and Care Services, 2023c, p. 7). Beyond mental health and health-friendly choices, active aging is also framed instrumentally to enable older people to live safely and independently at home for as long as possible, to postpone the need for health and care services and to allow for the more efficient use of healthcare personnel and collective resources. The white paper contrasts bad with good health behaviors, underscoring the impact of lifestyle choices on overall health (Norwegian Ministry of Health and Care Services, 2023c, p. 21). Negative behaviors such as smoking, poor diet, lack of physical activity, and harmful substance use are associated with increased risks of illness and functional impairment. Conversely, adopting good health behaviors can significantly reduce these risks and support the goal of living safely at home (Norwegian Ministry of Health and Care Services, 2023c, p. 21).

Framed as a health-friendly choice, the concept of active aging implies that older adults have the agency to choose an active and socially inclusive lifestyle or, conversely, to become inactive and socially excluded. Although this framing stresses individual agency, the white paper also emphasizes society's responsibility to reassess its perception of older people and to foster an understanding that recognizes older adults not as burdens, but as individuals with valuable contributions and resources (Norwegian Ministry of Health and Care Services, 2023c, p. 47). Staying active and healthy promotes independence, self-sufficiency, and equal participation in society alongside younger individuals, thus underscoring the value of integrating older adults as vital members of the community (Norwegian Ministry of Health and Care Services, 2023c, p. 48).

Choosing to be active and healthy would thus include limiting the use of alcohol and substances and following recommended physical activity of at least 150–300 min of moderate intensity exercise or 75–150 min of high intensity exercise per week (Norwegian Ministry of Health and Care Services, 2023a, p. 56). In addition, it involves participating in cultural activities and/or volunteering. As such, active aging also becomes both an implicit and explicit strategy for combatting challenges connected to substance use in old age. From this perspective, harmful substance use represents a significant risk to active aging, ultimately undermining the opportunities to harness the contributions that older adults can offer to society. Therefore, promoting active aging not only addresses the issue of substance use and its detrimental effects on older adults, but also maximizes the valuable resources and contributions that older adults can provide to society.

### Mobilization Against Loneliness

The Plan reveals that loneliness is prevalent particularly among individuals over 80 years old. In 2015, three out of 10 older people aged 80 years and older reported feelings of loneliness (Norwegian Ministry of Health and Care Services, 2015, p. 38). In older age, loneliness can arise from various changes in living situations, such as retirement, divorce, declining health, and the loss of friends and family members. Furthermore, loneliness among older adults can lead to harmful substance use, exacerbate health conditions, and create difficulties in managing daily life (Norwegian Ministry of Health and Care Services, 2015, pp. 38–39).

In earlier white papers on care for older people, loneliness was often discussed in close connection with social isolation. For example, the 2014 white paper juxtaposed loneliness with social support, defining the latter as “receiving love and care, being respected and valued and belonging to a community” (Norwegian Ministry of Health and Care Services, 2014, p. 39). The example further revealed that social networks promote health because they contribute to social support. In this context, loneliness implies social exclusion and isolation, which could be caused by poverty, stigma, mental health issues, and problematic substance use (Norwegian Ministry of Health and Care Services, 2023b). A more recent white paper, however, distinguishes more clearly between loneliness and social isolation as follows: “social isolation is an objective and quantitative indicator of lack of social contact,” whereas loneliness is more subjective, such that “you can feel lonely even if you have a rich social life and you don’t have to feel lonely even if you don’t” have a rich social life (Norwegian Ministry of Health and Care Services, 2023a, pp. 74–75). Both social isolation and loneliness are identified as risk factors in the white papers, as they can contribute to, and be exacerbated by, problematic substance use as summarized in the following excerpt:

High alcohol intake also increases the risk of weakened social networks and social problems such as relationship breakdowns, loss of friends and unemployment. Increased alcohol use among older people can exacerbate health problems and be a sign of dissatisfaction and loneliness. (The Norwegian Ministries, 2017, p. 16)

Given these links between loneliness, social isolation and substance use, and as Norway's older population continues to grow, addressing loneliness in this demographic has become an urgent public health priority (Norwegian Ministry of Health and Care Services, 2023c). Solutions to loneliness include preventing passivity and isolation and reducing harmful substance use that may arise from a lack of social contact and support (Norwegian Ministry of Health and Care Services, 2014, p. 39).

Volunteer organizations have also been identified as beneficial with respect to the prevention of loneliness. On the one hand, volunteer organizations could recruit older people to engage in volunteering initiatives to meet others and build social networks (Norwegian Ministry of Health and Care Services, 2014, p. 40). On the other hand, volunteer organizations could engage older individuals in social activities such as dancing and going for walks (Norwegian Ministry of Health and Care Services, 2015, p. 38). Such initiatives could lead to improved mental and physical health outcomes, helping combat the damaging effects of loneliness at the same time as promoting a more active lifestyle. Encouraging older adults to remain active and socially engaged can significantly enhance the overall health and quality of life of this population. This approach corresponds to the broader goals of active aging and can reduce the likelihood of individuals turning to substances as a means of managing loneliness or stress (Norwegian Ministry of Health and Care Services, 2015).

### Preventive Home Visits

A third strategy mentioned in the Plan was preventive home visits. It was indicated that guidelines would be developed to strengthen the competency of first-line health workers, particularly in addressing key health-related risk and protective factors among older people. A preventive home visit guide was prepared in 2017, and, among other things, home care nurses were recommended to discuss substance use, drug use, social community, and physical activity during home visits to older people (Norwegian Ministry of Health and Care Services, 2018; 2019, p. 83). Preventive home visits were described as follows:

… part of the overall preventive work aimed at elderly people in the municipality and are an offer of advice and guidance to elderly people who do not have services, or who have limited services from the municipality. (Norwegian Ministry of Health and Care Services, 2018, p. 131)

A multitude of reasons underpins these visits, centred on both supporting older people's independence and reducing pressure on the health and care system, as outlined in the white papers. The aims include utilizing individuals’ own resources to meet care needs, prolonging residence at home and delaying institutionalization, strengthening independence and mastery of daily life, and reducing the need for health-care services (Norwegian Ministry of Health and Care Services, 2023c, p. 7; The Norwegian Ministries, 2021, p. 38). Taken together, these multifaceted objectives underscore the importance of preventive home visit as a measure to promote good health at the same time as easing the overall burden on health and care services.

This strategy corresponds with both national directives and global recommendations, such as those from the Organisation for Economic Co-operation and Development (OECD), to strengthen early intervention services for individuals with mild to moderate substance use problems, especially older adults (Norwegian Ministry of Health and Care Services, 2015, p. 17; OECD, 2014, p. 117). By identifying issues early, providing holistic care, and connecting older people with appropriate resources, preventive home visits play a crucial role in promoting healthier choices and preventing the escalation of health problems (Norwegian Ministry of Health and Care Services, 2015). This proactive approach not only benefits the individual, but also contributes to the long-standing policy goal of more sustainable and effective use of the resources of the Norwegian health care system (Norwegian Ministry of Health and Care Services, 2014).

Preventive home visits are further exemplified and supported by the ABC of Elder Care initiative. Established in the early 2000s, the ABC of Elder Care is designed to increase the quality of care for older adults by providing continually updated and reflective knowledge on best practices for municipalities. This initiative aligns with national directives, regulations, and laws aimed at improving the wellbeing of older adults (The Norwegian National Centre for Ageing and Health, 2021). In recent years, substance use has been integrated as an important element of the ABC of Elder Care training approach, described similarly by two white papers (The National Strategy on Alcohol and Community and Mastery) as follows:

In the ABC of Elder Care, substance use among elderly people is thematised, and the goal is for employees who have completed the training to have discussed attitudes towards substance use among elderly people, have knowledge of the risky use of substances among elderly people and be able to raise awareness and motivate service recipients to make preventive and health-promoting choices. (Norwegian Ministry of Health and Care Services, 2023c, p. 87; The Norwegian Ministries, 2021, p. 38)

In summary, preventive home visits are expected to address not only substance use problems, but also the intertwined issues of loneliness and inactivity among older people. Extending the policy discussions on combating loneliness and promoting active and healthy aging, preventive home visits carry an expectation that older people will make certain, specific lifestyle choices. These include engaging in cultural and communal activities such as dance clubs and physical exercise, as well as minimizing alcohol and substance use. Preventive home visits have thus become important channels through which older people can be “helped” to make good, health-promoting choices that conform to state priorities around early intervention in harmful substance use in old age.

## Discussion

We have outlined three proposed strategies – active aging, mobilization against loneliness, and preventive home visits – that constitute Norway's approach to addressing substance use issues among older adults. Collectively, these strategies emphasize early intervention and a strong focus on prevention rather than treatment. These strategies are based on the premise that older adults are independent and autonomous individuals with valuable resources to contribute to society. Recognizing their self-determination, these approaches assert that older adults can address their own substance use challenges, enabling them to engage fully in community life. Furthermore, these strategies aim to optimize the use of limited health care resources, thereby supporting sustainable welfare provisions. Below, we examine the scope of these approaches in addressing the diverse needs of older adults and explore how they extend beyond substance use to inform government solutions for older-age care challenges.

### Social Inequalities in National Strategies for Substance Use in Old Age

Researchers have advocated for multidisciplinary approaches that target individual substance use in old age at the same time as incorporating social and environmental determinants of health (Beynon, 2009; Blanco & Lennon, 2020; Chhatre et al., 2017). This recommendation is based on increased diversity in terms of income, culture, gender, sexuality, and religion, reflecting multifaceted needs among new older people (Foster & Walker, 2015; Heinonen et al., 2023; Westwood, 2019). For example, in Norway, socioeconomic differences have been associated with access to social networks and support systems and the likelihood of developing health problems (Norwegian Ministry of Health and Care Services, 2014, p. 40; 2019, p. 17; 2023a, p. 75; 2023c, p. 21). Older people with low levels of education and access to fewer resources have been described as more likely to be lonely and to develop health problems. Preventive home visits, for example, have been reported to increase the utilization of home-based care among highly educated older people (Bannenberg et al., 2021). This effect has been attributed to the empirical assumption that older people with higher levels of education are more likely to have better access to care services and to be more expressive about their health and needs to visiting care professionals. Given that substance use in old age can further complicate health needs and service navigation (Bergh et al., 2021; Fredwall & Antonsen, 2023), these socioeconomic and educational inequalities are likely to be exacerbated among older adults who use substances. Consequently, older adults who both lack social and economic resources and use substances are at particular risk of unmet care needs because they may have fewer capacities and opportunities to seek, access, and negotiate appropriate care, a condition often referred to as “care poverty” (Kröger, 2022).

Socioeconomic differences in substance use can also be understood through the alcohol harm paradox, which observes that disadvantaged groups suffer greater alcohol-related harm than advantaged groups despite similar or lower levels of consumption (Boyd et al., 2022). This paradox highlights that vulnerability to substance use disorders is multifactorial, including biological, psychological, geographic, sociological, political, and economic determinants, rather than merely a function of consumption. Similarly, white papers recognize that alcohol use is greatest among individuals with higher socioeconomic status, although its negative consequences are more prevalent in groups with lower socioeconomic status (Norwegian Ministry of Health and Care Services, 2019). Prevention strategies such as promoting active aging and reducing loneliness and social isolation may therefore help reduce harmful substance use. However, interventions that create social spaces for older adults (e.g., activity programmes or social clubs) can unintentionally increase alcohol consumption if drinking becomes a normative part of participation. In this context, promoting active aging and mobilization against loneliness might not be a univocal solution to the perceived problem.

A significant perspective illuminated in white papers is the reconceptualization of older people as a resource rather than a burden to society (Norwegian Ministry of Health and Care Services, 2023c). This perspective substantiates research indicating that, when older adults participate in discussions about care services, service providers obtain better information to develop adaptive strategies for older people (Vestby et al., 2017). Although older people are recognized as capable, self-autonomous, and independent, we argue that the proposed approaches still tend to view those who use substances as vulnerable and careless. The strategies considered in this context – active aging, combating loneliness, and preventive home visits – appear to be designed for an older adult population that is capable, resourceful and motivated. However, this notion of “the active older adult” does not necessarily extend to those struggling with substance use issues.

When addressing these populations more explicitly, a subtle yet important shift seems present in the white papers, from “the capable older adult” to “the substance-using older adult framed as responsible for poor choices”. This recharacterization is evident in white papers that contrast beneficial and harmful health behaviors, which strongly underlines lifestyle choices. For example, as the Plan states, “With good lifestyle habits such as social, physical, and cognitive activity, as well as good nutrition, older people can enjoy more healthy years of life. In this context, it is important to monitor developments in older people's use of alcohol and other intoxicants, perhaps especially in the transition from working life to retirement” (Norwegian Ministry of Health and Care Services, 2015, p. 39). Similarly, the 2023 white paper on Community and Mastery observes that “health behaviours and lifestyle habits such as smoking, physical activity, diet and substance use have a major impact on health. Good health behaviours and healthy lifestyles can reduce the risk of illness” (Norwegian Ministry of Health and Care Services, 2023c, p. 21). By foregrounding individual lifestyle change, these texts frame substance use as a problem of personal responsibility. Consequently, municipal health and care services are urged to proactively address substance use and to encourage older people “to make preventive and health promoting choices” (Norwegian Ministry of Health and Care Services, 2023c, p. 87). When health is primarily framed as the outcome of individual lifestyle choices, substance use is implicitly moralized as a failure of self-control or responsibility. A paternalistic and moralistic tone attends these recommendations as follows: they shift the burden of prevention onto individuals and prioritize behavior-change approaches rather than addressing structural or system-level determinants of substance use. This emphasis leaves little room for recognizing how factors such as poverty, isolation or limited service access shape substance use in older age.

White papers do, however, recognize the increasing diversity among older adults and the varied profiles of individuals who engage in substance use. They acknowledge the different stages of substance use and the broad spectrum of needs within this demographic (Norwegian Ministry of Health and Care Services, 2012, p. 73; Norwegian Ministry of Social Affairs, 2003, p. 21). This understanding is largely absent from the strategies designed to address substance use among older adults. A notable gap is the absence of clear guidelines for implementing active aging initiatives that accommodate the diverse needs of this population. Older adults with mobility issues or health complications often face significant barriers to participation in traditional programs (Tian et al., 2023), which may not be designed with their limitations in mind. The approaches under discussion still fail to meet individuals where they are, and they need to provide inclusive options that encourage participation regardless of physical ability.

Current solutions tend to focus on individuals who are at the onset of substance use problems, which means that older adults who do not participate in initiatives such as antiloneliness programmes or who lack access to preventive home visits may unintentionally be excluded from support, even though they might benefit substantially. For example, preventive home visits are largely available to individuals aged 80 years and above, with younger individuals receiving them only upon recommendation from their GP (Bannenberg et al., 2021), implying that older adults who fall in between service thresholds are at risk of unmet needs. Preventive home visits may therefore fail to fulfil the Plan's objective of expanding awareness and screening for substance-use problems (The Norwegian Ministry of Health and Care Services, 2015, p. 21). Home-based services, rightly promoted to support aging in place and to reduce thresholds for accessing care (The Norwegian Ministry of Health and Care Services, 2023c), might nevertheless be unable to meet the needs of younger older adults experiencing substance use disorders, particularly those whose situations do not fit the standard entry points for services.

Moreover, the “problem definition” emphasizes the transition from working life to retirement (2015) and the suggested strategies are focused heavily on prevention (The Norwegian Health and Care Services, 2015, p. 39). Because the problem is framed primarily as preventing new problems in the transition to retirement, older people in drug-assisted rehabilitation (DAR) and those with long-term alcohol use are largely left outside the scope of these strategies. This is particularly concerning because the number of people aged 60 years and above in DAR has reportedly tripled since 2015 (The Norwegian Health and Care Services, 2023c, p. 24). Research has indicated that older DAR patients and older adults with chronic alcohol problems often experience shame, guilt, and internalized stigma, which contribute to social isolation, smaller social networks, and limited support from family and friends (KORFOR, 2015; Liahaugen Flensburg et al., 2025). These experiences suggest that such groups could benefit substantially from initiatives that mobilize against loneliness and promote active aging. The limited consideration of this group in prevention-focused strategies points to important gaps in how social and health equity are addressed within the welfare state.

### Substance Use Prevention Strategies in Old Age as “Governing Techniques”?

The framing of solutions to substance use problems in old age, such as addressing loneliness, inactivity, and preventive home visits, could be more usefully understood as “techniques of government” (Foucault, 1991; Lemke, 2002; Lorenzini, 2023) that enable agendas to roll back welfare provisions in the care of older people (Ågotnes et al., 2022; Blix & Ågotnes, 2023; Jacobsen, 2017) rather than directly addressing substance use issues in old age. These “techniques of government” are not neutral: by prioritizing prolonged engagement in working life, drawing on older adults’ own resources, delaying residential care, and promoting self-reliance among older people, they advance solutions to demographic and fiscal challenges of an aging population in Norway (Foster & Walker, 2015). A similar pattern is evident in Finland and Sweden, where following the implementation of active aging policies, older adults felt that social welfare no longer safeguarded their health (Nyqvist et al., 2022).

In the Norwegian context, these governing logics become visible in the organization of preventive home visits and home care services. Discussions on preventive home visits have shown that, although the initiative began as a strategy to improve the quality of life for older people, it transitioned into a way of delaying the need for long-term care and facilitating sustainable welfare provisions (Bannenberg et al., 2021). In addition, home care providers operate under stringent regulations set by municipal authorities that dictate the types of care they are permitted to deliver. This regulatory framework typically involves assessments that categorize service users on the basis of specific needs, leading to a standardization of care that privileges clinical and physical aspects over social or holistic considerations (Brenne et al., 2024). Home care workers are often assigned narrow tasks and limited time slots for each client, which reduces the flexibility needed to adapt care to the unique contexts of individual service users (Wollscheid et al., 2013). In this sense, when home care providers are constrained by narrowly defined roles that exclude substance use discussions or interventions, they are unlikely to identify or address these issues even when they are evident in their clients.

Government machinations and attempts to govern are based on a deep-seated understanding of the object to be governed (Bacchi & Goodwin, 2016; Ball, 2015; Rose & Miller, 2010, p. 179). Congruent with this line of argument, the agendas designed to address substance use among older adults can be read as reflecting an embedded perception of this demographic as careless. As discussed earlier, policy texts that foreground lifestyle choices and personal responsibility contribute to constructing substance-using older adults as having made poor or ill-informed decisions regarding their health and well-being, and perhaps as lacking full capacity to make such decisions (Norwegian Ministry of Health and Care Services, 2023c, p. 21). Here, as previously mentioned, we see a paradoxical disconnect between an “active aging” informed approach and a perhaps more moralistic view of substance use. The framing of older substance users as “flawed” simplifies the governance process, allowing policymakers to impose solutions that may fit preconceived notions of what older adults need rather than what they actually require.

Approaches such as active aging and preventing loneliness, which often include recommendations for physical activity, social engagement, and educational initiatives around substance use, aim to empower older adults by encouraging proactive health management. Simultaneously, they cast a shadow of judgement over those who struggle with substance use issues. This moralizing tendency reinforces the framing of older substance users as “flawed” subjects who have failed to live up to the expectations of active, self-managing aging. The indirect message is that if older adults adhere to these guidelines, then they can implicitly manage or prevent substance use challenges on their own. This expectation disregards the complex realities of addiction, which often do not align with simplistic solutions predicated on self-management (Henden, 2023).

This prevention-centred, self-management-oriented framing is also associated with a particular temporal assumption that substance use problems among older adults develop exclusively in old age. Such a narrow understanding essentially neglects the experiences of older adults who have grappled with alcohol dependence or addiction earlier in life and may continue to face these challenges as they age. Preventive approaches to substance use among older adults can inadvertently exclude those who would benefit most from harm reduction strategies, treatment programmes, and integrated care models. Neglecting the diverse realities of substance use among older adults, ranging from long-term addictions to situational substance use, risks perpetuating cycles of marginalization, where individuals are left without adequate resources or support systems. Therefore, national strategies that do not accommodate the full spectrum of substance use experiences and trajectories effectively marginalize older adults who require targeted support and intervention, thereby undermining commitments to social and health equity in older-age care.

## Methodological Limitations

This study focused on three of Bacchi's questions (Q1, Q2 and Q5) rather than on the full six-question suite; that choice sharpened attention to meanings, assumptions and effects. However, this work inevitably narrowed the analytic scope and may have missed additional analysis of the historical antecedents, silences in the problem definition, and arenas where the problems have been disseminated that the omitted questions would have revealed. Similarly, WPR analysis focuses on the processes through which social problems are identified and framed in particular ways, prompting scholars to remain analytically reflective rather than claim direct causal connections between policy representations and practical solutions (Rönnblom & Edwards, 2025). Consequently, applying this method does not by itself generate concrete service outcomes for substance-using older adults.

Some limitations stem from our data selection and handling. Older adults’ substance use is rarely treated as a standalone issue and is usually discussed within broader policy domains (public health, elder care, and drug policy), which complicates the isolation of the phenomenon. The analysis used only national-level white papers published between 2003 and 2024. This selection strategy may omit insight into local, clinical or volunteer organizations’ perspectives and practice-level data that could materially alter problem representations.

The initial NVivo coding and subsequent translation of codes into English using DeepL introduced the potential for coding biases and subtle meaning shifts; although the codes were read iteratively to mitigate this, machine translation and single-language-source reliance remain sources of interpretive uncertainty.

Finally, analytic choices about inclusion might limit drawing conclusions beyond the Norwegian national policy context. Eighteen white papers were screened, but only seven provided sufficiently substantive content to be analyzed in depth; the exclusion of eleven papers, although noted and cited when relevant, might still have led to missed nuances. Future studies could address these limitations by triangulating national-level document analysis with local policy documents and service-level data generated from qualitative interviews.

## Conclusions

This paper underscores the critical role that national policies play in the process of shaping the discourse around substance use problems among older adults in Norway. While highlighting strategies to eliminate loneliness, promote active aging, and implement preventive home visits, the current policy framework portrays older adults as capable and autonomous but simultaneously vulnerable when faced with substance use issues. Moreover, the solutions do not recognize diversity and tend to categorize older adults as a single homogeneous group. This approach contradicts evidence of the growing diversity among older adults and obscures the complexities surrounding substance use. Such approaches may exacerbate health inequalities for older individuals from low socioeconomic backgrounds and marginalize those with long-term substance use histories. Advocacy for a more nuanced understanding of aging and substance use that considers the diverse needs and strengths of older adults may be needed. Future policies should prioritize comprehensive solutions that not only encourage early intervention, but also provide robust treatment and rehabilitation options for long-term users, thereby fostering a more inclusive and equitable approach to addressing substance use issues in older populations.
